# Cryogenic Reliability Evaluation of Glass Fabric–Reinforced Composites Using Novel Slip-Prevention Method

**DOI:** 10.3390/ma16010121

**Published:** 2022-12-22

**Authors:** Yong-Cheol Jeong, Dong-Ha Lee, Seul-Kee Kim, Jeong-Hyeon Kim, Jae-Myung Lee

**Affiliations:** 1 Department of Naval Architecture and Ocean Engineering, Pusan National University, Busan 46241, Republic of Korea; 2 Hydrogen Ship Technology Center, Pusan National University, Busan 46241, Republic of Korea

**Keywords:** cryogenic reliability, LNG cargo containment system, flexible secondary barrier

## Abstract

Glass fabric–reinforced composites are the main insulating material components of the secondary barrier of cargo containment systems (CCSs), because they prevent liquefied natural gas (LNG) leakage during transport. Nevertheless, it is difficult to evaluate the material performance of glass fabric–reinforced composites at cryogenic temperatures (−163 °C) because it takes approximately 7 days to prepare the test specimens and because the slip-based test frequently fails. Although glass fabric–reinforced composites for the secondary barrier of LNG CCSs show various structural vulnerabilities, enhancing their material performance is significantly limited owing to the reasons mentioned above. This study evaluated the structural vulnerabilities and failure characteristics of glass fabric–reinforced composites by using the slip-prevention test method to determine the level difference and adhesive vacancies. The failure surface and the thermal expansion of the composites were also observed, to analyze their mechanical characteristics. By adopting our proposed test procedure, the failure rate of the experiment decreased by approximately 80%, and the sample preparation time for manufacturing was significantly shortened, to 1 day.

## 1. Introduction

Natural gas has received significant attention as an environmentally friendly fuel because its combustion emits less carbon dioxide than conventional fossil fuels do [1]. Hence, its demand as a fuel has gradually increased in many industries, such as automotive and shipbuilding. Liquefied natural gas (LNG), which is natural gas cooled to a liquid state, at approximately −163 °C, is the most effective and preferred method for shipping and storing natural gas, because its volume is approximately reduced 600 times compared with its gaseous state. Hence, structural reliability and thermal reliability at cryogenic temperatures are the most important functional requirements for LNG storage systems, as they require complex multilayered insulation systems to maintain temperatures such as −163 °C.

There are two main structural classifications for LNG cargo containment systems (CCSs): independent and membrane [2,3]. Independent tanks are self-supporting and separate from the hull, whereas membrane tanks are not autonomous and consist of a thin layer (membrane) supported by insulation [4,5]. Membrane tanks are preferred in the market for being more cost-effective and having significantly larger storage capacity than their independent counterparts. Depending on the insulation system adopted for the CCS, membrane tank designs can be classified into two types, Mark III and NO96; the former type is widely employed for LNG CCSs because of its higher efficiency [6].

**Figure 1:**
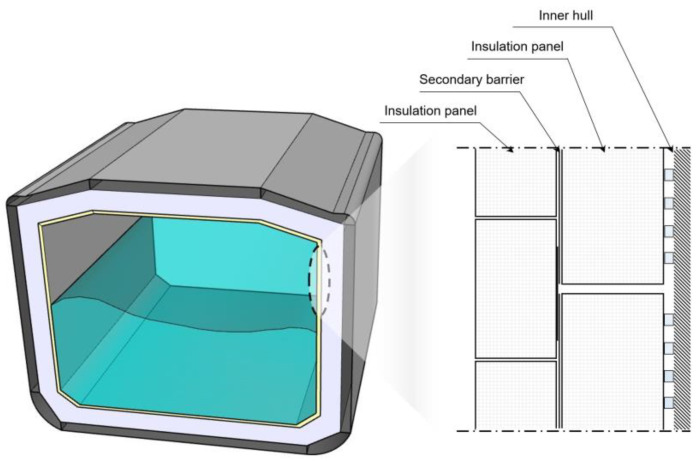
Mark III–type LNG insulation system.

Figure 1 schematically illustrates a Mark III–type LNG insulation system. Its surface is covered with a thin layer of cryogenic temperature-resistant 304 L austenitic stainless steel, known as the primary barrier, that prevents LNG leakage. Further, the corrugated structure of the plate prevents thermal contraction and expansion [7]. The inside of the membrane panel contains two-layered reinforced polyurethane foam (R-PUF), embedded with a secondary barrier [8,9] to seal the tank against gas leakage for 15 days after the primary barrier has been fractured.

**Figure 2:**
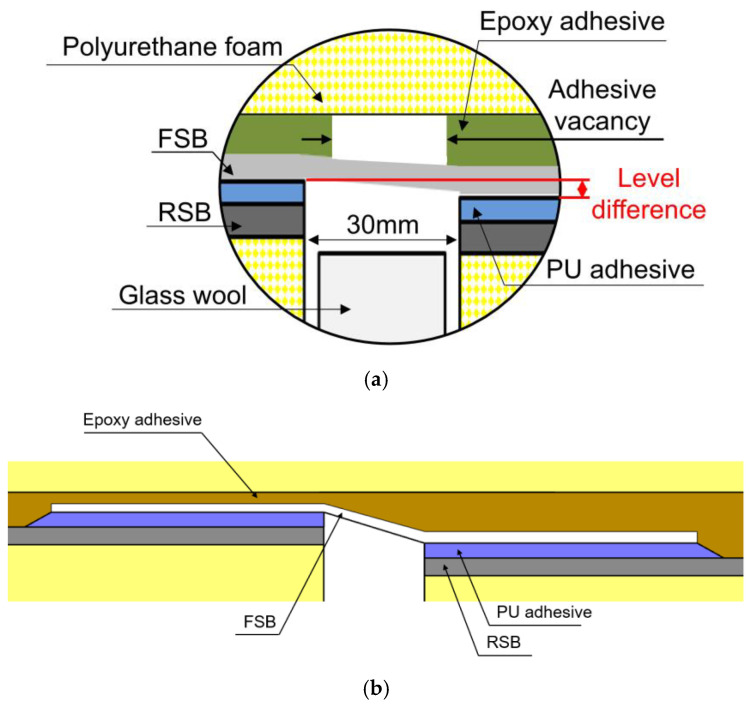
Schematic composition of (**a**) a secondary barrier in Mark III–type LNG insulation systems and (**b**) variation of the mechanical characteristics caused by the level difference phenomenon.

Figure 2a illustrates the schematic composition of the secondary barrier in Mark III–type LNG insulation systems. There are two main concerns: the adhesive vacancy and the level difference. Adhesive vacancy can occur from the bonding process, whereas a level difference can occur from differences in manufacturing tolerances during the manufacturing process of the LNG insulation panel. The secondary barrier contains flexible and rigid sections, respectively known as the flexible secondary barrier (FSB), comprising glass fabric, rubber, and aluminum foil, and the rigid secondary barrier (RSB), comprising glass fabric and aluminum [10,11]. The flexibility of the secondary barrier is required because of its subjection to thermal loads under cryogenic temperatures and bending loads under hogging and sagging. The bottom insulation panel is typically attached to the RSB with a 30 mm gap to allow for thermal deformation. Subsequently, each RSB is attached to the FSB with polyurethane (PU) adhesive, and the FSB is attached to the top bridge pad with epoxy adhesive. The R-PUF and FSB components exhibit a level difference owing to their disparate manufacturing tolerances during the manufacturing process of the LNG insulation panel [12,13], which is defined as uneven and curved parts of up to 3 mm between the insulation panels, owing to the nonuniform flatness of the inner hull of the vessel. Figure 2b is the exaggeratedly drawn variation of the FSB caused by the level difference phenomenon. The occurrence of a level difference can likely weaken the performance of the FSB because it can be bent, resulting in tensile load and stress concentration in the FSB. Hence, the mechanical properties of an FSB should be investigated on the basis of variations in the level difference.

**Figure 3:**
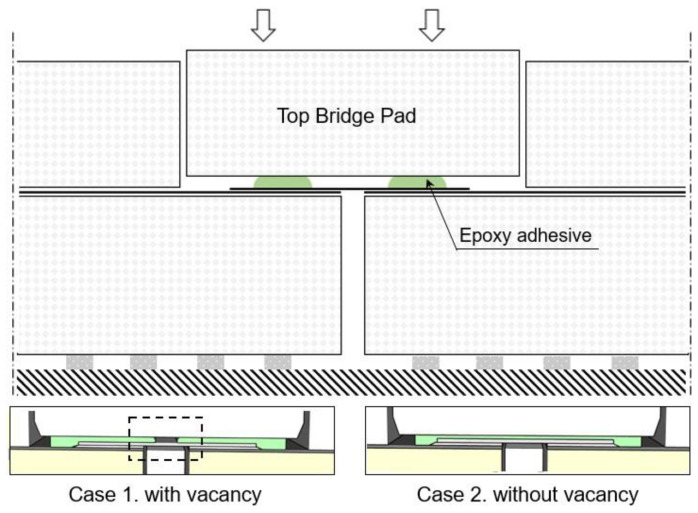
Schematic illustration of the manufacturing (bonding)-process-dependent adhesive vacancy in Mark III–type LNG insulation systems.

Figure 3 shows a schematic of the bonding-process-dependent adhesive vacancy of the insulating system. Epoxy adhesive was applied to the top bridge pad in two or three lines during the tank manufacturing process. The top bridge pad is bonded to the FSB by using a pressure pad. Subsequently, the epoxy adhesive is spread onto the end of the FSB; however, the epoxy adhesive does not reach the middle of the FSB, because of its swift hardening or insufficient quantity. Hence, the mechanical properties of the FSB should be investigated on the basis of variations in the adhesive filling ratio because various types of vacancies might emerge between the FSB and the top bridge pad.

Over the past decades, several studies have reported on the mechanical properties of glass fiber–reinforced composites, which are similar to the secondary barrier in LNG CCSs [14,15,16,17]. Oh et al. investigated the fatigue performance of an LNG insulation system containing a newly developed metallic secondary barrier, which showed improved tightness and strength over that of conventional secondary barriers [18]. Additionally, many researchers have reported the mechanical properties of adhesively bonded joints as secondary barriers [19,20]. For example, Lee et al. studied the debonding failure characteristics of epoxy- and polyurethane-based adhesives at cryogenic temperatures. Furthermore, they qualitatively investigated the debonding failure damage pattern from ambient to cryogenic temperatures. Additionally, the material behavior of the multilaminate bonding system was precisely analyzed on the basis of the stress–strain relationship [21].

Although several researchers have studied the mechanical behavior of the secondary barrier, only a few studies have considered the effects of the level difference on adhesively bonded joints. Yoon et al. reported on the influence of the curing pressure and e-glass fabric density on the bonding performance of adhesive joints with a 3 mm level difference at cryogenic temperatures [22,23]. Nevertheless, the simultaneous investigation of the mechanical performance of FSBs with a measurable level difference and adhesive vacancies is completely absent. This study evaluates the structural vulnerabilities and failure characteristics of a glass fabric–reinforced composite, where the influences of the level difference and the adhesive vacancies were considered by employing a novel slip-prevention test method. Parallelly, the failure surface and thermal expansion degree of the composites were meticulously observed to evaluate their mechanical performance.

## 2. Experimental Section

### Proposed Mechanical Test Method

**Figure 4:**
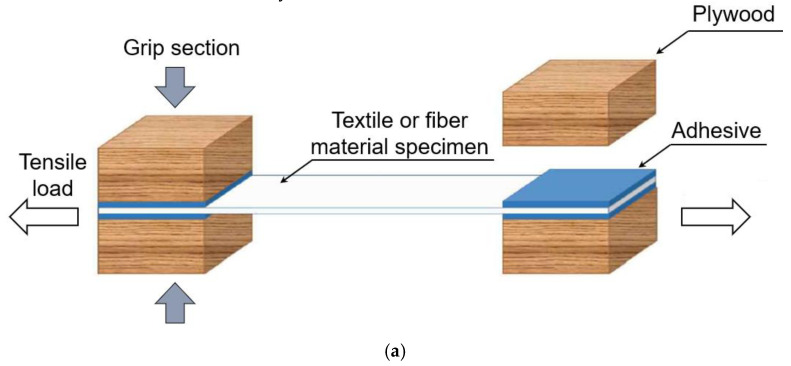

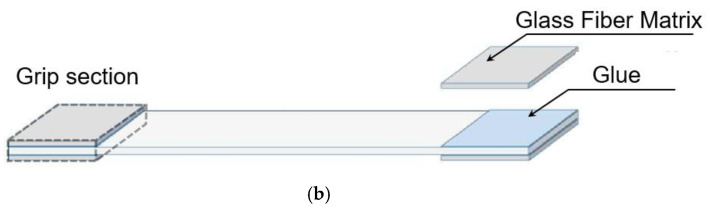
Schematic illustration of the preparations of the testing samples: (**a**) conventional method and (**b**) proposed method.

**Figure 5:**
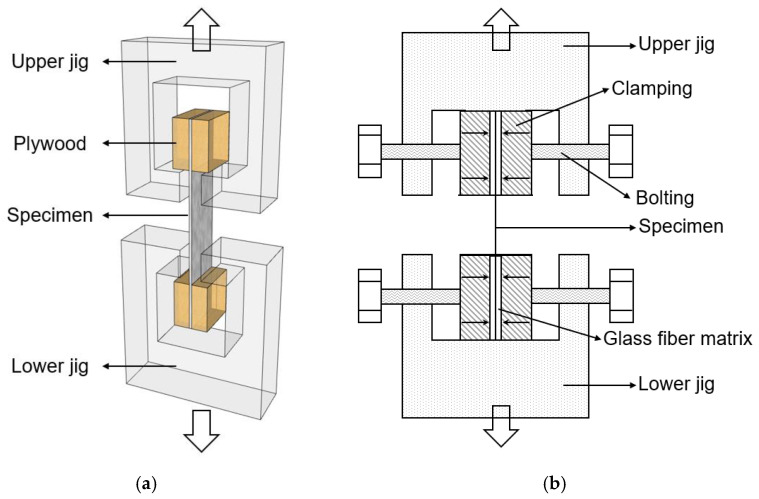
Schematic illustration of the preparations of the experimental setup: (**a**) conventional method and (**b**) proposed method.

The mechanical test of the secondary barrier for the MARK III type was conducted by using GTT M 3101 (Gaztransport & Technigaz, Chevreuse, France). Figure 4 and Figure 5 show illustrations of the preparations of the testing samples and experimental setup, respectively. In the conventional test method, the grip section of the textile/fiber is bonded to the plywood with a PU adhesive. However, fractures occasionally occur in the adhesive joints at the grip section prior to the fracture of the specimen, and slippage can also occur between the jig and the specimen at the grip section. Particularly, slippage occurs at cryogenic temperatures, making accurate measurements impossible. Furthermore, the curing time for the PU adhesive is 7 days, as stated in the conventional test standard.

To overcome these problems, we proposed a novel mechanical test method. The specimen is initially bonded to a glass fiber matrix by using a cyanoacrylate adhesive and then clamped with a machine bolt. Stiff clamping prevents the rotation of the specimen parts after matrix cracking, ensuring a nearly uniform stress distribution over the cross section. Furthermore, the curing time for the cyanoacrylate adhesive is only 1 day.

Stefanov et al. reported a dynamic mechanical analysis of carbon black-filled cyanoacrylate adhesive bulk films. Cyanoacrylate adhesives require shorter curing times for reaching a solid state than do other adhesives, while possessing good thermal properties at cryogenic temperatures, because gelation during phase separation maintains the morphology in place [24]. Therefore, in our work, compared with the conventional test method, the premature fracturing at the adhesive joints and slippage of the specimen at the grip section were attenuated because of the cyanoacrylate adhesive, owing to its excellent thermal properties and enhanced grip section. Furthermore, the conventional test method required an adhesive curing time of 7 days, whereas the proposed test method required only 1 day. Therefore, fractures did not occur at the adhesive joints under cryogenic conditions. Moreover, a large deformation did not occur under a high tensile load, resulting in accurate fracture unit values, better strength, and a high success rate, confirmed by the decrease of the test failure rate to 20%. Thus, the experimental reproducibility and reliability of our novel tensile test method and its ability to control the fracture location parameters were verified.

**Figure 6:**
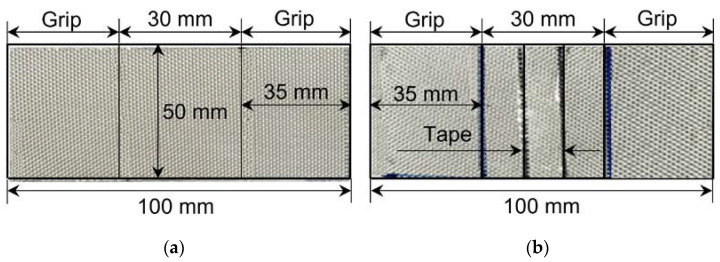
Shapes and dimensions of the mechanical test specimens: (**a**) without an adhesive filling vacancy and (**b**) with an adhesive filling vacancy.

Experimental samples were prepared to investigate the effects of the level difference and the adhesive filling ratio of the secondary barrier. The FSB (Huntchinson, Paris, France) and R-PUF panel (KANGRIM INSULATION, Changwon, Korea) were mechanically bonded using an epoxy adhesive with a density of 120 kg/m^3^ to produce a conventional insulation structure. All specimens for the mechanical tests were fabricated according to the strip test standard ISO 1421 [25]. The gauge length was set to 30 mm, which is the distance between the bottom insulation panels. The total length, width, and thickness of the specimens were 100, 50, and 0.7 mm, respectively (Figure 6). The total bonding lengths between the FSB and R-PUF panels from the edges of the gauge length were 15 mm (adhesive filling ratio of 50%), 21 mm (adhesive filling ratio of 70%), and 30 mm (adhesive filling ratio of 100%). To induce an adhesive filling vacancy, the middle of the FSB was taped to prevent adherence to the R-PUF, with tape lengths of 15, 9, and 0 mm. The bonding area of the FSB determined the adhesive filling ratio of the specimen. Furthermore, the adhesive vacancy was positioned in the middle of the FSB to replicate actual performance when the top bridge pad is attached with two or three lines of epoxy glue. After bonding, the specimen was compressed with 10 kg of weight for a week to cure the adhesiveness of the epoxy.

Plywood layers of various thicknesses were inserted in each specimen to induce various level differences. Specimens with a level difference of 3 and 6 mm were fabricated using plywood with 6T and 12T, respectively, because of the 5.6 mm level difference of the LNG CCS insulation panel [26]. The plywood had dimensions of 30 × 50 mm to adjust the jig joint size. The jig joint of each specimen adhered to the strips to prevent slippage during the tensile tests.

There are several different mechanical tests in the ISO standards for individual strips; however, no appropriate test standard exists for strips with a level difference. Hence, we designed a special tensile test with symmetrical specimens to verify the tensile performance of a secondary barrier with a level difference. Furthermore, mechanical tests were also performed on specimens with a 0 mm level difference, following the ISO 1421 strip test standard, while the designed symmetrical tensile test was performed on specimens with a level difference. The tensile tests were conducted at a crosshead speed of 5 mm/min and at −120 °C, which is the temperature that the secondary barrier of the MARK III tank is typically exposed to in the field. Table 1 lists the parameters used for the tensile tests.

**Figure 7:**
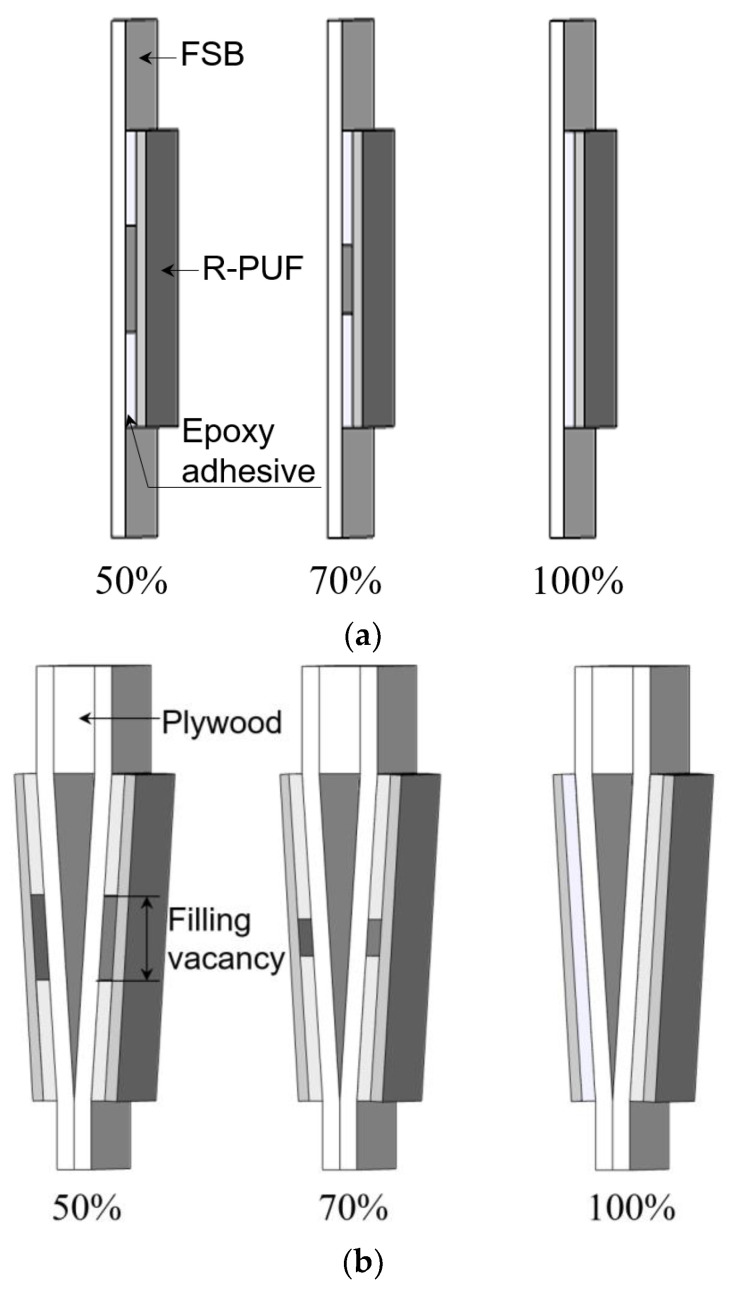

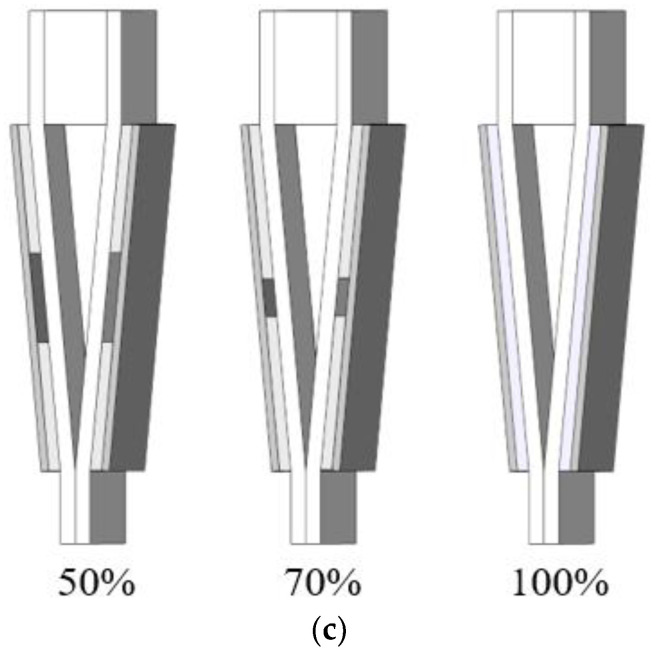
Schematic diagram of specimens with different level differences/adhesive filling ratios: (**a**) 0 mm/50%, (**b**) 3 mm/70%, and (**c**) 6 mm/100%.

Figure 7 shows the schematic diagram of specimens with different level differences/adhesive filling ratios. Although the FSB is an anisotropic material, only the tensile load direction generated by hogging and sagging was considered in this study, owing to the shrinkage of the two insulation panels in the opposite direction. The test was performed on five specimens to ensure the validity of the results. The average of three main values was accepted, apart from the maximum and minimum values. Moreover, the measured ultimate load was divided by two in the symmetrical mechanical tests, to determine the ultimate load of the FSB.

**Figure 8:**
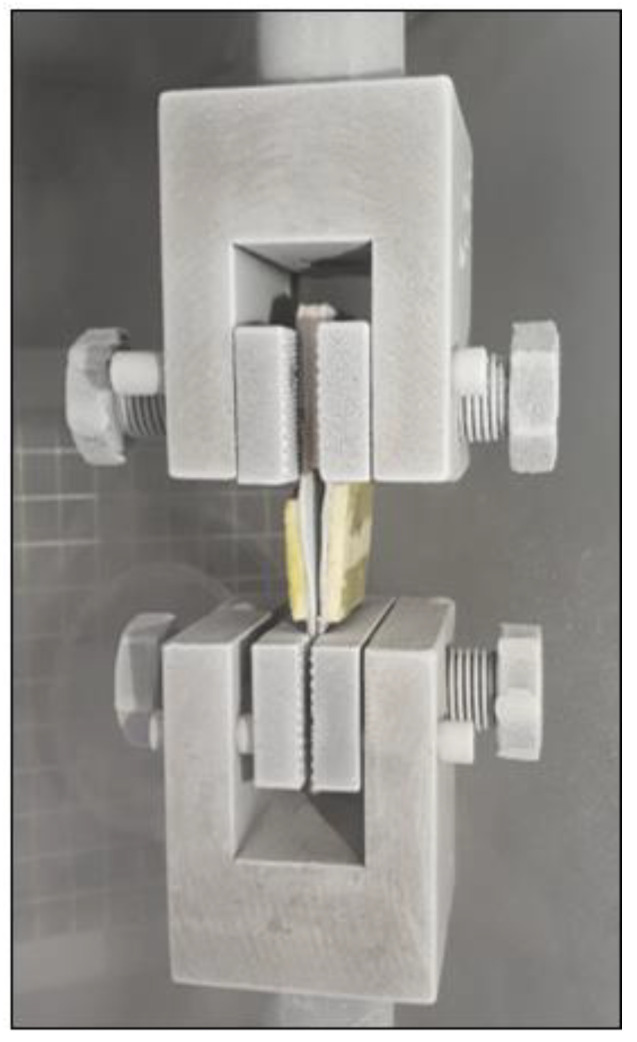
Photograph of the UTM apparatus used for the mechanical tests.

The mechanical tests were conducted by using a universal testing machine (UTM; KSU-5M, Kyung Sung, Ansan, Korea) connected to a high-pressure liquid nitrogen container to investigate the mechanical properties and fracture points of the FSB in a MARK III tank. The UTM was used with a cryogenic insulation chamber, as shown in Figure 8. Liquid nitrogen (−196 °C) was distributed throughout the insulation chamber to maintain the desired temperature. Furthermore, the cryogenic mechanical test specimens were cooled until their temperature matched that of the surrounding environment before conducting the mechanical tests.

The mechanical load and displacement were measured by using a constantly moving crosshead of the UTM. Although the LNG was stored at −163 °C, the operating temperature of the FSB was −120 °C, owing to the insulation materials; hence, all cryogenic tests were conducted at −120 °C [27]. The target temperature of −120 °C (the temperature faced by secondary barriers during operation) was maintained using a thermometer and a control system in the cryogenic chamber. Furthermore, the liquid nitrogen was circulated by a fan to realize a uniform distribution of heat.

The thermal stress generated during cooling was eliminated by controlling the jig position. The mechanical behavior was observed through the window of the cryogenic chamber, owing to the variable mechanical behavior of the glass fibers in the FSB.

## 3. Results and Discussion

### 3.1. Maximum Tensile Load and Reliability

Table 2 presents the maximum fracture loads and displacements of the tensile test specimens related to their level difference and adhesive filling ratio at −120 °C. Overall, the fracture load and displacement increased and decreased, respectively, with increasing the adhesive filling ratio. However, the fracture load and displacement both decreased with an increase in the level difference.

The maximum tensile load of the tensile test specimen with a level difference of 0 mm and an adhesive filling ratio of 100% was 2.7% and 14.3% higher than those of the specimens with adhesive filling ratios of 70% and 50%, respectively. However, the maximum tensile loads of the specimens with a level difference of 3 mm and an adhesive filling ratio of 100% were 7.1% and 11.7% higher than those of the specimens with adhesive filling ratios of 70% and 50%, respectively. Finally, a level difference of 6 mm and an adhesive filling ratio of 100% led to maximum tensile loads that were 6.7% and 8.8% higher than those of the specimens with adhesive filling ratios of 70% and 50%, respectively.

Furthermore, the maximum tensile load of the tensile test specimen with an adhesive filling ratio of 50% and a level difference of 0 mm was 8.8% and 9.3% higher than those of the specimens with a level difference of 3 and 6 mm, respectively. Accordingly, the maximum tensile load for the specimen with an adhesive filling ratio of 70% and a level difference of 0 mm was 2.6% and 10.0% higher than those of the specimens with a level difference of 3 and 6 mm, respectively. However, the maximum tensile load for the specimen with an adhesive filling ratio of 100% and a level difference of 0 mm was 11.5% and 14.8% higher than those of the specimens with a level difference of 3 and 6 mm, respectively. The percentage increase in the maximum tensile load of the specimens with respect to their level difference and adhesive filling ratio strongly indicated that both these factors influence the maximum tensile load of the FSB.

It can thus be concluded that an FSB with a level difference of 6 mm and an adhesive filling ratio of 50% is the most prone to failure, with a high probability of fracture occurring in the middle. On the other hand, the most stable conditions are when the FSB has zero level difference and a 100% adhesive filling ratio; in this case, any fracture is likely to manifest at the edge of the FSB.

### 3.2. Effect of the Level Difference

**Figure 9:**
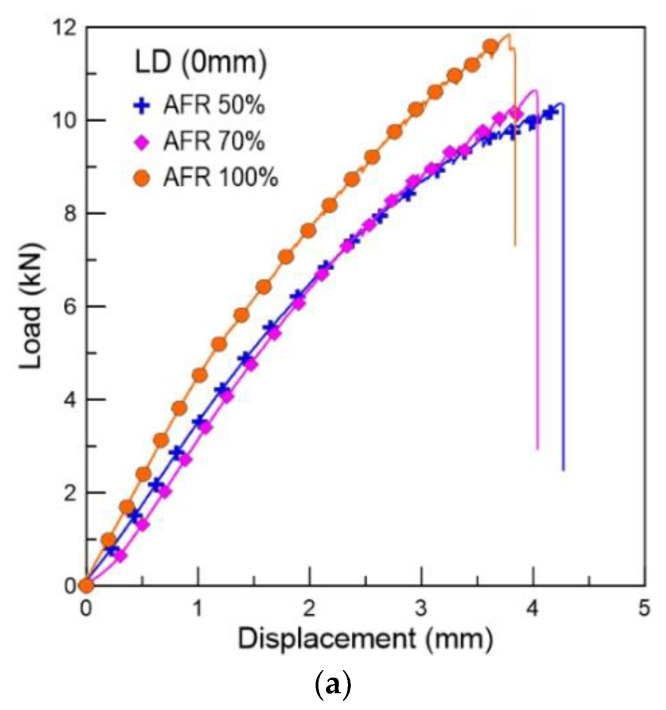

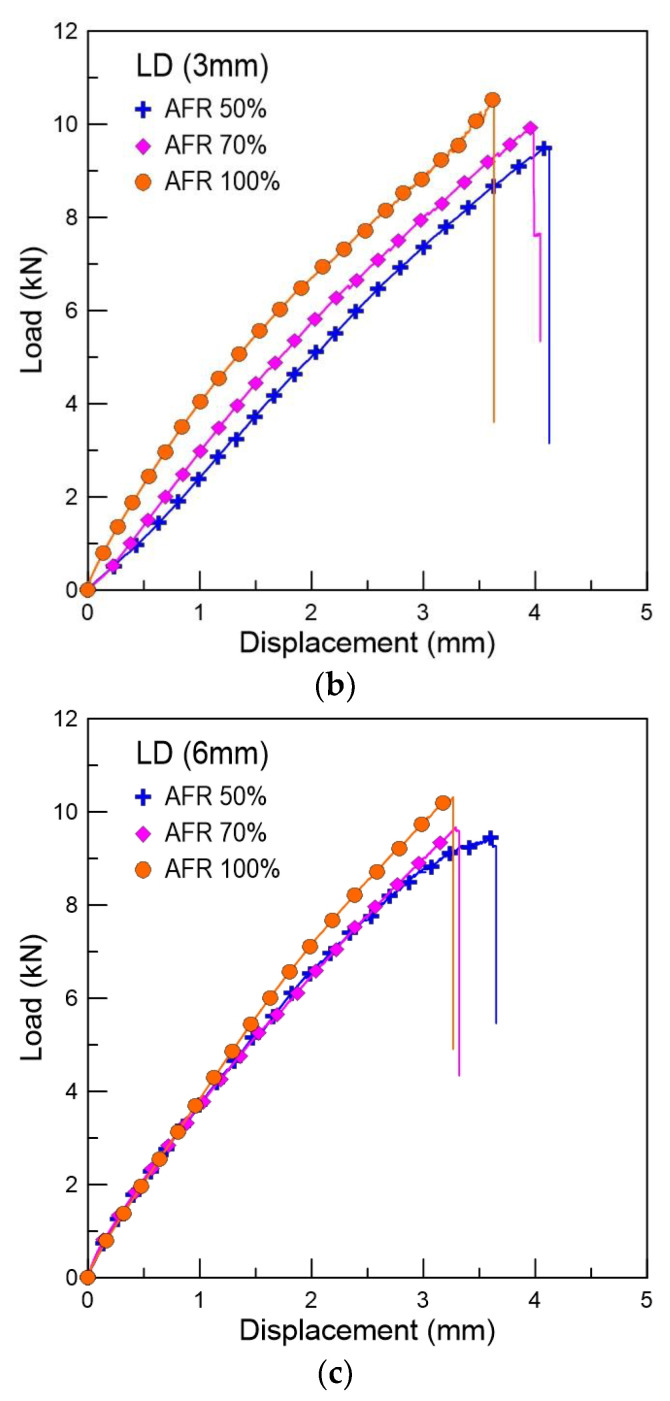
Tensile load and displacement curves of specimens with various level differences: (**a**) 0 mm, (**b**) 3 mm, and (**c**) 6 mm.

**Figure 10:**
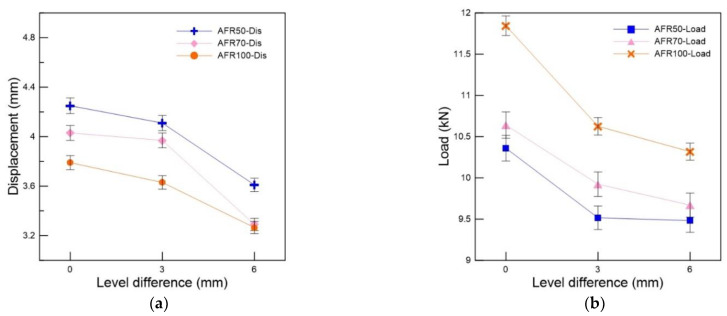
Variation in the (**a**) maximum displacement and (**b**) maximum load of tensile specimens with varying adhesive filling rations in relation to the level difference.

FSB fractures in the field originate from several causes, including impacts, thermal loads, and structural issues; however, tensile loads are the most common cause, as previously mentioned. Therefore, in this study, tensile tests were performed on the FSB specimens to investigate the effect of the level differences and adhesive vacancies. Figure 9 shows the load-displacement curves for the specimens according to the level difference at −120 °C. These curves show that the displacement and load decreased as the level difference increased. The specimen with a level difference of 3 mm exhibited a more compact degradation than that with a level difference of 0 mm (Table 2). However, the specimen with a level difference of 6 mm exhibited a severe degradation: its fracture load was 11.85 kN when the level difference was 0 mm with an adhesive filling ratio of 100%; furthermore, the fracture load was 10.32 kN, with a level difference of 6 mm and an adhesive filling ratio of 100%. The tensile properties of the specimens with a level difference of 6 mm and an adhesive filling ratio of 100% were 12.9% lower than those with a level difference of 0 mm and the same adhesive filling ratio. This indicated that a level difference above 6 mm critically promotes an FSB’s susceptibility to fracturing because of structural issues. This behavior stems mainly from the stress concentration, which also affects the fracture location. Figure 10 shows that the stress concentration deteriorated with an increase in the level difference of the specimens. Overall, a level difference of 6 mm and above was inferred to significantly increase the probability of an FSB to sustain failure during operation, owing to bending- and thermal-derived tensile stress.

### 3.3. Effect of the Adhesive Filling Ratio

**Figure 11:**
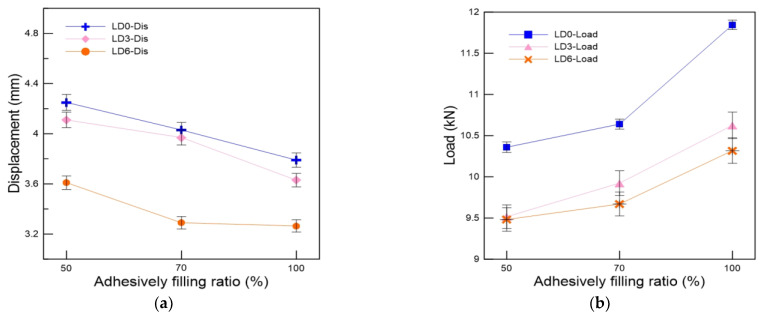
Variation in the maximum (**a**) displacement and (**b**) tensile load of the tensile test specimens according to the adhesive filling ratio.

Figure 11 shows the typical load-displacement variations of the tensile test specimens according to the adhesive filling ratio at −120 °C. A decrease in the adhesive filling ratio increased the tensile fracture load and decreased the tensile fracture displacement. The specimen with an adhesive filling ratio of 100% exhibited significantly larger mechanical reinforcement than that with an adhesive filling ratio of 50%. Accordingly, the specimen with an adhesive filling ratio of 70% had more compact reinforcement than that with an adhesive filling ratio of 50%. Furthermore, the specimen with an adhesive filling ratio of 50% and a level difference of 0 mm exhibited a tensile fracture load of 10.36 kN, whereas that of the specimen with an adhesive filling ratio of 100% and a level difference of 0 mm exhibited one of 11.85 kN. The tensile fracture load of the specimens with an adhesive filling ratio of 100% and a level difference of 0 mm was 14.3% higher than those with an adhesive filling ratio of 50% and the same level difference. This suggested that the adhesive filling ratio critically affects the occurrence of fractures in FSBs. Therefore, adhesives should be applied to 100% of the FSB surface to minimize the fracturing probability.

**Figure 12:**
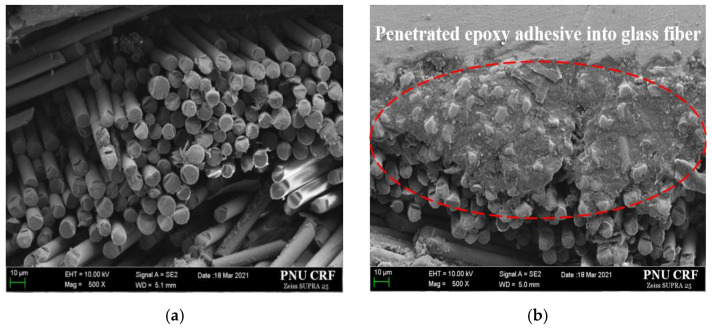
SEM images of the fracture surface of tensile test specimens: (**a**) surface of glass fibers in nonadhesive-treated FSB; (**b**) epoxy adhesive-penetrated glass fibers of FSB.

Figure 12a shows the scanning electron microscopy (SEM) images of the fracture surface of the FSB, verifying its glass fiber composition; furthermore, it shows a bundle of glass fibers at the fracture surface of an FSB specimen. Figure 12b shows the impregnated epoxy adhesive penetrating the glass fibers of the FSB. It was assumed that the impregnated epoxy adhesive acted as an epoxy coating, absorbing the tensile load rather than the FSB while hardening the glass fibers on the surface. Additionally, the stress in the FSB was dispersed and transmitted to the epoxy matrix through the epoxy resin chain. Therefore, the epoxy adhesive effectively improved the tensile strength of the FSB by reducing the stress concentration at the defects while increasing the maximum tensile fracture load [28]. Also, the adhesive-penetrated FSBs possessed higher surface energy and work values than their untreated equivalents [29], suggesting that applying the epoxy adhesive on the FSB surface strongly promoted crack healing. The penetrated epoxy can be used as a surface treatment, reinforcing the tensile strength of the FSB by absorbing the tensile load [30].

### 3.4. Fracture Location within Specimens

**Figure 13:**
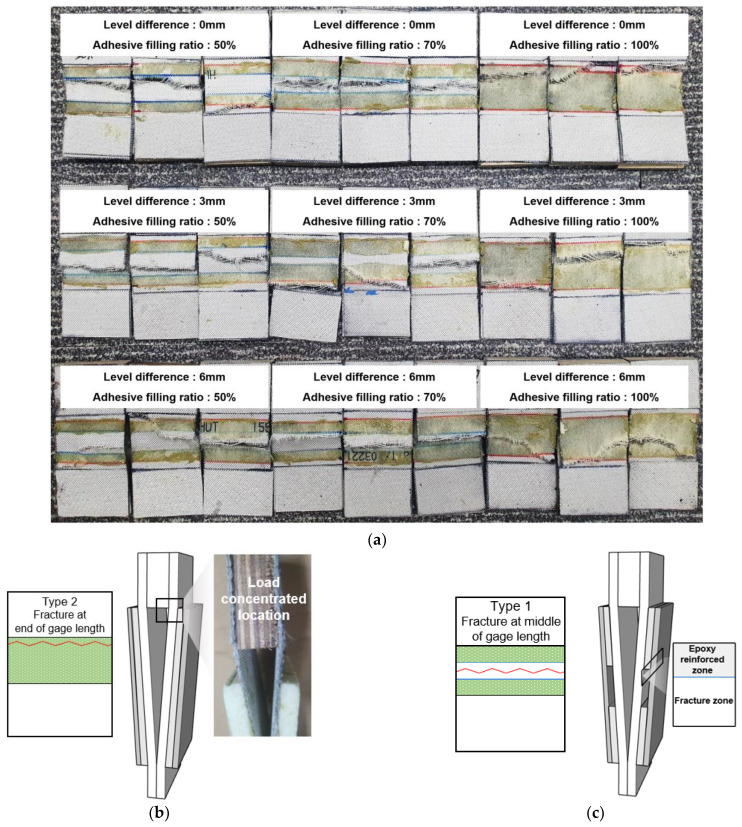
Fracture location in specimens according to the level difference and adhesive filling ratio: (**a**) images of tensile test specimens; schematic diagrams of a fracture (**b**) in the middle and (**c**) at the end of the gauge length.

Fractures generally occurred near the weaker points of our FSB specimens and were influenced by two main parameters: the level difference and the adhesive filling ratio. Figure 13 shows the fracture characteristics according to the level difference and the adhesive filling ratio of the specimens. Two main fracture types were observed. The first was located at the end of the gauge length of the specimen, with a level difference of 6 mm and an adhesive filling ratio of 100% (Figure 13b). In general, specimens with an adhesive filling ratio of 100% were subjected to a uniform tensile load; however, those with samples were subjected to a nonuniform tensile load at the location where the FSB bent. The second fracture type occurred in the middle of the gauge length of the specimens, with a level difference and adhesive vacancies (Figure 13c). In this case, the tensile load was concentrated in the nonadhesive-covered areas of the FSB (if the adhesive was not applied to the entire surface) because the impregnated epoxy adhesive acted as a surface coating that provided a reinforcement effect.

The location of the fracture was determined on the basis of the level difference and adhesive filling ratio of the specimens. For the specimens that featured both a level difference and an adhesive filling ratio, the fracture occurred in the middle of the gauge length. Therefore, the fracture location was more strongly influenced by the adhesive filling ratio than by the level difference.

## 4. Conclusions

This study evaluated the structural vulnerability and failure characteristics of glass fabric–reinforced composites at cryogenic temperatures (−120 °C) while factoring in the influence of the level difference and adhesive vacancies, using a newly developed test procedure with slip-prevention features. The main results are summarized as follows:

- We proposed a mechanical test method for textile and glass fiber–reinforced materials under cryogenic conditions. The jig was machined bolted and clamped with the specimen, preventing the rotation of the grip section of the specimen.
- Slippage and the premature fracture of the adhesive joints prior to the specimen were prevented by using our newly proposed test method. The curing time was reduced to 1 day, and the rate of experimental failure decreased to 20%.
- As a result of the tensile test with the proposed jig and specimen, fracturing was more likely to occur at the end of the gauge length in specimens with a level difference. Parallelly, the maximum tensile load and displacement decreased with the increasing level difference.
- Fracturing was more likely to occur at the middle of the gauge length in specimens with adhesive vacancies because of the general load concentration. Concurrently, the maximum tensile load and displacement increased and decreased, respectively, with the increasing adhesive filling ratio.
- A microstructural analysis revealed that the fracture load increased with the increasing adhesive filling ratio because the impregnated epoxy adhesive absorbed the tensile load rather than the FSB and hardened the glass fibers on the surface.

Consequently, our proposed test method is highly suited for conducting tensile tests on textile and glass fiber–reinforced materials under cryogenic conditions, by significantly reducing the experimental failure rates and preventing slippage to offer accurate measurements. Furthermore, we observed that compared with the level difference, the adhesive filling ratio of the specimens more prominently deteriorated the fracture load and determined the fracture location. Therefore, scrupulous inspections for adhesive vacancies should be performed during the manufacturing management stage to ensure the cryogenic reliability of the FSB.

## Figures and Tables

**Table 1:** Mechanical test scenarios for the FSB.

Material	Temperature	Level Difference	Filling Ratio
FSB	−120 °C	0 mm	50%
			70%
			100%
		3 mm	50%
			70%
			100%
		6 mm	50%
			70%
			100%

**Table 2:** Mechanical properties of the FSB under each condition.

Material	Temp (°C)	Level Difference (mm)	Filling Ratio (%)	Fracture Load (kN)	Displacement (mm)
FSB with epoxyadhesive	−120	0	50	10.36	4.25
			70	10.64	4.03
			100	11.84	3.79
		3	50	9.51	4.11
			70	9.92	3.97
			100	10.63	3.63
		6	50	9.48	3.61
			70	9.67	3.29
			100	10.32	3.27

## Data Availability

The data presented in this study are available on request from the corresponding author.
